# Real-World Analysis of Adherence to Abemaciclib and Endocrine Therapy in Women with HR+/HER2− Breast Cancer

**DOI:** 10.3390/biomedicines13030546

**Published:** 2025-02-21

**Authors:** Maria Rosaria Valerio, Federica Martorana, Maria Vita Sanò, Daniela Sambataro, Gianmarco Motta, Lucia Motta, Giuliana Pavone, Vittorio Gebbia, Giuseppa Scandurra

**Affiliations:** 1 Medical Oncology Unit, Policlinico Paolo Giaccone, University of Palermo, 90133 Palermo, Italy; mariarosaria.valerio@unipa.it; 2 Department of Clinical and Experimental Medicine, University of Catania, 95124 Catania, Italy; federica.martorana@humanitascatania.it (F.M.); gianmarco.motta@humanitascatania.it (G.M.); lucia.motta@humanitascatania.it (L.M.); giuliana.pavone@humanitascatania.it (G.P.); 3 Medical Oncology Unit, Humanitas Istituto Clinico Catanese, Misterbianco, 95045 Catania, Italy; mariavita.sano@humanitascatania.it; 4 Medical Oncology Unit, Ospedale Umberto I, 94100 Enna, Italy; daniela.sambataro@unikore.it; 5 Medical Oncology Unit, Cdc Torina, 90146 Palermo, Italy; 6 Medical Oncology, School of Medicine, University of Enna Kore, 94100 Enna, Italy; giuseppa.scandurra@unikore.it; 7 Medical Oncology Unit, Ospedale Cannizzaro, 95126 Catania, Italy

**Keywords:** abemaciclib, breast cancer, adherence, barriers, dose-intensity, drug claims

## Abstract

**Background:** Adherence to oral anticancer therapies among breast cancer patients is an often-overlooked issue. A lack of patient compliance can be caused by several factors, and may hinder the efficacy of prescribed medication, leading to a shorter than expected survival. In this context, few data about adherence to CDK4/6 inhibitors in real-world practice are available. We report here the results of a retrospective analysis of adherence to abemaciclib plus endocrine therapy in a cohort of women with hormone receptor-positive (HR+), epidermal growth factor 2 negative (HER2−) breast cancer.** Methods:** Abemaciclib adherence was computed as the ratio between the total number of cycles/months that medication was supplied and the months between the first and the last prescription. The proportion of Days Covered (PDC) ranged from 0 to 1. A score of 0.8 (i.e., 80% adherence rate) was the cutoff used to classify the patients as adherent (0.8 ≤ PDC ≤ 1) or non-adherent (0 ≤ PDC < 0.8). The received dose intensity was also calculated. **Results:** The abemaciclib pharmacy claims of 100 women with HR+/HER2− breast cancer were retrieved. Most patients (91%) were treated in the advanced setting. Abemaciclib was more frequently taken with an aromatase inhibitor (63%) than with fulvestrant (27%). In this population, the adherence rate was high (92.25% + 1.939 SD). The proportion of non-adherent patients taking abemaciclib with PDC <0.8 was 12%. There was a significative correlation between the occurrence of side effects and the use of <5 drugs for non-oncological illnesses, probably reflecting concomitant non-oncological diseases. **Conclusions:** Adherence to abemaciclib-based therapy is high in a real-life setting, pending the adequate and proactive management of patients. The careful evaluation of patients and detailed information about expected adverse events are essential to ensure adherence to this antineoplastic agent.

## 1. Introduction

In recent years, novel oral anticancer therapies have greatly improved the survival of patients with solid tumors, including breast cancer (BC) [1]. Achieving and maintaining adequate adherence rates to these treatments is pivotal to obtaining optimal anticancer effects, eventually resulting in favorable clinical outcomes [2]. Adherence is a complex multifactorial issue, and its optimization may be challenged by side effects, schedule complexity, and emotional and psychological factors, including the patient’s motivation, understanding of the disease, and support system [3,4].

The issue of compliance for oral agents in BC is not new, and adherence to oral hormonal therapy (HT) in hormone-positive (HR+)/human epidermal growth factor 2 negative (HER2−) BC patients may be lower than expected or perceived by prescribers due to a variety of barriers [5,6,7,8]. Regular HT intake is essential for achieving the best possible outcomes in terms of disease control since it prevents cancer from progressing by continuously inhibiting the targeted enzymes [8,9]. However, HT represents a long-term commitment for patients, who often experience side effects and an impaired quality of life [6]. Two systematic literature reviews of behavioral and educational interventions to improve HT adherence reported no significant improvements compared to usual care [10,11]. The authors reported variability in the terminology and definitions of adherence to medication used, which may create biases and render comparisons among different studies difficult. 

Recently, the addition of a cyclin-dependent kinase 4/6 inhibitor (CDK4/6i) (i.e., palbociclib, ribociclib or abemaciclib) to HT became the standard of care for HR+/HER2− metastatic BC patients, and two of these agents (ribociclib and abemaciclib) also showed efficacy in the adjuvant setting [1,12,13,14]. Among CDK4/6i, abemaciclib (ABE) is particularly active in metastatic endocrine-resistant patients, achieving an invasive disease-free survival advantage in the adjuvant treatment of high-risk hormone-sensitive BC [12,14,15,16].

However, treatment with ABE may induce significant gastrointestinal side effects, mainly diarrhea and fatigue, which may interfere with treatment adherence [17]. Several strategies can be adopted to improve compliance with this agent, including the proactive management of side effects, educating patients and caregivers, simplifying oral polypharmacy, or implementing support systems through healthcare providers, family or external groups [18,19]. Still, limited data about compliance to CDK4/6i, including ABE, are available in real-world scenarios.

Here, we report a retrospective multicentric analysis of treatment adherence in a real-life series of patients receiving ABE plus HT for HR+/HER2− BC.

## 2. Materials and Methods

*Study design*. This non-interventional, retrospective analysis was conducted at five medical oncology units, one academic hospital, two cancer centers, and two general hospitals. Due to the study’s non-interventional, retrospective nature and the use of fully anonymized data, informed consent was waived. 

*Data sources*. According to Italian regulations, novel anticancer agents subjected to monitoring by the Italian Medicine Agency (Agenzia Italiana per il Farmaco, AIFA, Roma, Italy) must be prescribed via a web-based application [20]. This platform allows the production of three-weekly or monthly drug claims that record prescription dates, patients’ characteristics, the drug dose (including potential dose reductions), toxicity, laboratory tests, and clinical responses. Drug claims are e-referred to the pharmacy every four weeks before there is a drug shortage to avoid treatment interruptions. Previous prescription discharge on the electronic platform is a prerequisite for giving the green light to the new one. Toxicities leading to drug dose reductions are electronically reported to the regional pharmacovigilance office. For all-time prescriptions, medical oncologists send drug claims electronically to the nearest public community pharmacy. Public community pharmacies are organized according to a hub-and-spoke model on a geographical basis. A central pharmacy acts as a hub coordinating all the spoke offices. Public community pharmacists verify the appropriateness of prescriptions and send drugs to the patient’s address. This e-referral system was implemented by the local government’s public health system of the Pharmacy Service to avoid gatherings during the COVID-19 pandemic.

*Adherence measurement.* There is no universally standardized method for measuring medication adherence. The Pharmacy Quality Alliance is a standard set of measures employed to assess pharmacy performance using administrative claims data to calculate measure rates such as the medication possession ratio (MPR) and the proportion of days covered (PDC); these are two validated measures of adherence based on the percentage of days the patient has medication available, which, when used together, reduce the risk of adherence overestimation [21]. PDC is the method most often used to calculate medication adherence using prescription refill data from electronic records at the population level. It is defined as the proportion of days in the treatment period “covered” by prescription claims for the same medication or another in its therapeutic category. The PDC formula is (Number of days in the period covered)**/**(Number of days in period) × 100. The PDC is the level above which the medication is reasonably likely to achieve most of the potential clinical benefit (threshold of 80%) for many chronic conditions. Adherence was calculated and reported over a fixed 12-month treatment period, starting from treatment initiation and the first drug claim. A score of 0.8 (i.e., 80% adherence rate) was the cutoff used to classify the patients as adherent (0.8 ≤ PDC ≤ 1) or non-adherent (0 ≤ PDC < 0.8). The duration of follow-up was defined as the time in months from the first prescription issued by the pharmacist in the community pharmacy to the last prescription registered and the date the treatment was discontinued for any reason, including progressive disease, death, severe toxicity, or loss of follow-up. This was previously described in [22,23]. This measure without stratification requires a minimum denominator of 30 for reliability. If the minimum denominator size is met, the measure should not be used for performance measurement, including in accountability programs. Reliability testing, which was conducted on the MS Medicare data as a ratio of the signal-to-noise using the Adams beta-binomial reliability methodology, showed that the measure was reliable, with a mean reliability score of 0.70 [24]. To evaluate the potential impact of dose modifications on outcomes, the authors calculated the relative dose intensity (RDI), which considers drug interruptions and dose reduction over time. For the RDI calculation, the standard starting dose was set according to approved labels, i.e., abemaciclib 150 mg twice daily in combination with either letrozole or fulvestrant. The dose intensity was calculated using the Hryniuk and Busch methods [25,26]. The RDI formula is (total dose over the study period)**/**(standard total dose over the study period) × 100. Toxicity was graded according to the National Cancer Institute Common Terminology Criteria for Adverse Events (NCI CTCAE) v5., as previously described [27]. The correlation of the patients’ variables, including the type and severity of side effects and the number of drugs taken, with the adherence rate was analyzed.

*Statistics.* The patient’s clinical characteristics were reported as absolute numbers and relative rates with a 95% confidence interval, as needed. Correlation statistical analysis was performed, and graphs were generated using GraphPad Prism 10.1 (GraphPad Software, Boston, MA, USA), with two-tailed statistical significance levels set at p less than 0.05.

## 3. Results

### 3.1. Patients’ Population

**Figure 1:**
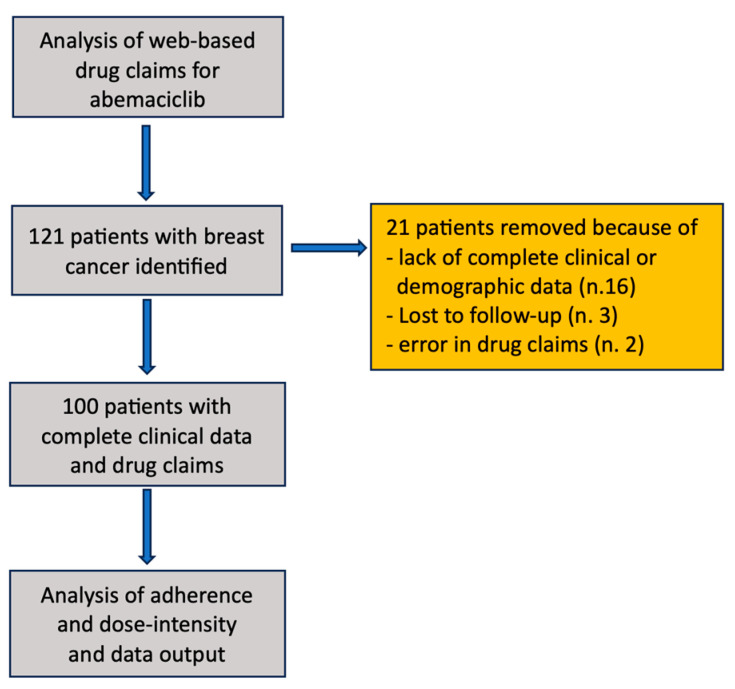
Study flowchart.

The study covered a period from 1 January 2018 to 31 December 2022. As shown in Figure 1, the analysis of web-based claims identified 121 patients with BC treated with abemaciclib. Twenty-one patients were removed from the analysis because of a lack of complete clinical and demographic data, errors in their drug claims, or because they were lost to follow-up. Therefore, the analysis included 100 patients treated with ABE plus an aromatase inhibitor (AI) or fulvestrant for advanced BC, or plus an AI as adjuvant postoperative therapy.

Table 1 summarizes the descriptive statistics of the patient characteristics and the adherence for each group of patients. Eligible patients had a median (range) age of 66 (30–90) years. Most patients (97%) had a 0*–*1 performance status according to the Eastern Cooperative Oncology Group (ECOG) scale. In total, 63 patients (63%) received ABE with letrozole and 27 (27%) received fulvestrant. Nine of the 100 patients were treated early, while the remaining 91 were treated in the advanced stage. In this setting, 23%, 18%, and 50% of the patients had bone-only, visceral, or soft tissue disease, respectively. Before ABE prescription, most patients were carefully screened for drug–drug interactions and for the presence of clinical characteristics indicative of severe diarrhea, such as inflammatory colorectal diseases, previous abdominal radiotherapy, colitis, diverticulosis, and polypharmacy [27]. Eighty-four patients started a full-dose therapy of 150 mg b.i.d., while 16 patients started at 100 mg/m^2^ b.i.d. due to the treating physician deciding to pursue a dose escalation strategy to prevent side effects (28). Polypharmacy, defined as >5 different medications being taken for diseases other than cancer, was recorded in 34 patients (34%).

### 3.2. Side Effects

Table 2 shows the side effects. Grade 3 or prolonged grade 2 diarrhea was recorded in 35% of cases and was the most frequent reason for dose reduction or treatment delay. Once treated adequately, no cases of permanent toxicity-driven ABE withdrawal were recorded. A time-trend analysis of the side effects showed that diarrhea was most frequent during the first two months of therapy and that its incidence subsequently dropped down to 3%. Grade 2*–*3 fatigue and anorexia emerged later and were reported in 21% and 16% of cases. Leukopenia was generally mild to moderate (grade 1*–*2), with reversible grade 3 events in 11% of cases. Thrombocytopenia was mild, with 15 patients (15%) reporting grade 1*–*2 toxicity. Grade 2 and 3 anemia was reported in 21 (21%) and 2 (2%) patients, respectively. No dose reduction was reported in patients starting with 100 mg b.i.d., even in those patients (n. 6) who had their dosage increased to 150 mg b.i.d. On the other hand, 21 patients (21%) treated with a starting dose of 150 mg b.i.d. required a reduction in their dose to 100 mg b.i.d. and 3 required a reduction in their dose to 50 mg b.i.d. Forty patients (40%) experienced some treatment delay, mostly for side effects (26%) or intercurrent non-cancer-related illnesses; in seven cases (7%), treatment was delayed due to claims delays caused by organizational dysfunction. The type of hormonal agent used, i.e.*,* AI or fulvestrant, did not influence the patients’ tolerance to ABE.

### 3.3. Adherence Rate

**Figure 2:**
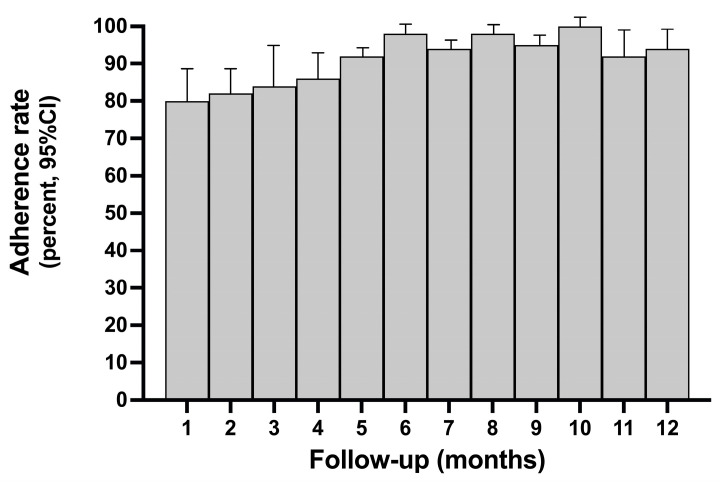
Monthly median adherence rate.

**Figure 3:**
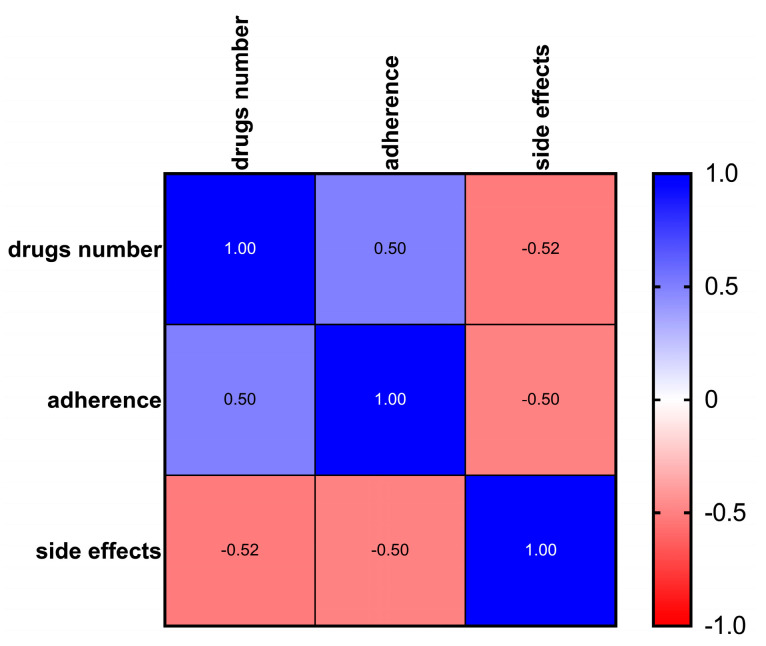
The heat map shows the correlation between the level of adherence, grade 2/3 side effects, and the use of <5 different medications for diseases other than cancer.

As shown in Table 2, the global adherence rate according to the PDC formula was 92.25% + 1.939 (SD), while the proportion of patients not adhering to PDC <0.8 was 12.5% (95% CI 6.2 to 20.9%). Figure 2 shows the time trend of the adherence rate during the prefixed follow-up time. Adherence was lower in the first 1*–*5 months but increased in the subsequent phases of follow-up. As shown in Figure 3, among patients treated with ABE, there was a significant correlation between the level of adherence, ≤5 different medications being taken for diseases other than cancer, and side effects (Pearson’s r ≤ 1). Patients taking <5 drugs for non-neoplastic diseases were more likely to achieve a high level of adherence to ABE, while those experiencing more intense side effects (grade 2*–*3) reported lower adherence. The occurrence of side effects probably negatively influenced adherence in the first three months of treatment, as inferred from the time trend of adherence, which shows lower adherence in the first three months of ABE followed by an increase after the dose reduction protocol, the better management of side effects and progress in the learning curve of patients and caregivers.

### 3.4. Dose-Intensity

As shown in Table 2, the programmed dose intensity, as per protocol, was 2100 mg/week (100%) of ABE in patients treated with a starting dose of 150 mg b.i.d., and 1400 mg/week in those who started at a dosage of 100 mg b.i.d. The received mean dose intensity was 88.7% (95% CI 81*–*94; mean 1988 mg/week) in the overall population. The mean received dose intensity was 82% (95% CI 73*–*89; mean 1968 mg/week) in patients initially treated with full-dose ABE at 150 mg b.i.d.; meanwhile, this value was 96.4% (95% CI 90*–*99; mean 1350 mg/week) in patients treated with 100 mg b.i.d.

## 4. Discussion

Abemaciclib is an orally active inhibitor of CDK4/6 that is indicated for HR+/HER2− BC patients, in combination with an AI or fulvestrant, depending on the clinical setting [1,12,13,14]. The most common adverse events associated with ABE are gastrointestinal toxicity, mainly diarrhea and fatigue, while cytopenia is less prevalent than with CDK4/6i [1,12,13,14]. This difference in the toxicity profile is most probably due to the ABE activity on different kinases [27]. Besides CDK4/6 inhibition, ABE also affects CDK9, which plays an essential role in intestinal cell proliferation. Moreover, abemaciclib exerts inhibitory effects on glycogen synthase kinase-3 beta (GSK3β) and Ca2+/calmodulin-dependent protein kinase CAMKII, which is involved in intestinal motility [27]. These antihormonal drug partners, i.e.**,** oral AI or intramuscular fulvestrant, do not influence the type and severity of ABE side effects [18,19].

As per other oral treatments, ABE non-adherence and high inter-individual pharmacokinetic variability can influence the response rates in metastatic settings. Adherence is even more critical in patients with surgically excised BC [13]. Adequate patient evaluation, education, and the proactive management of potential side events are crucial for optimizing adherence and the dose intensity [28]. These efforts represent a significant workload for oncologists dealing with BC. Therefore, insights into the rates of and barriers to adherence are important in clinical practice, considering the ever-increasing number of patients with access to ABE in the future [29].

The precise and reliable monitoring of adherence to antineoplastic drugs is challenging since different methods exist and many of them are only partly applicable to routine clinical practice [30,31,32]. Individualized therapeutic drug monitoring, the measurement of the plasma concentration of drug metabolites, and the electronic registration of daily drug assumptions have been used to monitor adherence and could potentially enable individualized treatment dosing [32].

There are few scientific reports about patients’ adherence to ABE in the medical literature. Smyth et al. conducted a retrospective real-world study that analyzed medical, and pharmacy claims for patients over three years [33]. This analysis included 454 patients with a 350-day follow-up who received ABE plus IA or fulvestrant, mainly for advanced-stage disease. One-quarter of the patients had received chemotherapy previously, and nearly half had received another CDK4/6i. Overall, 55% of patients had visceral metastases, with one-third having liver disease. Researchers reported an adherence rate of 88.7% for ABE, as determined by MDP.

Turcu et al. correlated the adherence of 330 patients with advanced BC to CDK4/6i with their age, gender, and follow-up duration [34]. The average follow-up period was 14.6 ± 12.5, 10.6 ± 7.1, and 8.6 ± 6.4 months for palbociclib, ribociclib, and ABE, respectively. The non-adherence rates for PDC were 12.8% with palbociclib, 14.6% with ribociclib and 14.7% with ABE. Overall, there was no significant correlation between adherence, age, or gender. However, a significant correlation was found with follow-up length. However, the relatively high adherence rate was calculated only on an 8-month interval, and essential information on the side effects leading to treatment discontinuation was incomplete.

A Canadian retrospective study on 65 patients reported the benefits of a patient-centered pharmacy practice according to the American Society of Clinical Oncology/National Community Oncology Dispensing Association standards and described its impact on CDK4/6i treatment use in locally advanced or metastatic breast cancer [28]. Such an approach ensures timely refills and strict monitoring and allows patients to achieve high adherence and persistence rates that are within the range reported in clinical trials. The mean PDC was 89.6%. However, only 14 patients received ABE. The treatment interruption rates were similar for palbociclib (63%) and ABE (64%). In the first three treatment cycles, 16% of patients treated with palbociclib had at least one dose reduction compared to 43% of patients treated with ABE. During the study period, 33% and 71% of patients had a dose reduction with palbociclib and ABE, respectively. Overall, the median time-to-treatment discontinuation was estimated to be 44.2 months in women treated with CDK4/6i + letrozole and 17.0 months in those treated with CDK4/6i + fulvestrant. The mean RDI was 85%, and the benefits of treatment were maintained regardless of the RDI levels.

We reported the results of a retrospective analysis of 100 women treated with ABE plus AI or fulvestrant as first-line treatment for metastatic disease or in an adjuvant postoperative phase. We found a high adherence rate with a relatively low proportion of non-adherence (92.25%). The time trend of adherence in the 12 months of observation shows lower adherence in the first 2–3 months of therapy with an increase in the following months, suggesting that side effects might have a significant influence; the ensuing dose reduction or better management may explain the improvement in adherence with time. Overall, there was no correlation between the patients’ adherence and menopausal status, socioeconomical level, comedication with AI or fulvestrant, or the clinic-pathological setting, such as dominant disease. The presence of a caregiver was not correlated, but almost all the women included had a caregiver, impairing the statistical analysis. On the other hand, there was a significant correlation between side effects and the use of <5 drugs (non-polypharmacy), the latter probably reflecting the number of concomitant non-oncological diseases [35,36]. The data align with those reported by other authors, confirming the high rate of adherence to ABE-based oral regimens. Our data confirm the results reported by other authors in terms of adherence [33,34,37]. However, there are significant differences compared to other experiences. Our data and previous experiences underline the importance of ensuring adequate patient information and management to prevent or manage non-adherence, while Turcu et al. reported 62 cases of ABE therapy with incomplete toxicity data [34]. We also report the monthly adherence rate, focus on the number of drugs used by patients for non-neoplastic diseases (polypharmacy versus <5 drugs) and consider the severity of side effects, which are the main determinant of poor adherence. Meanwhile, Turcu et al. focused on age and gender, with no significant association between these features, and adherence to CDK4/6i. Smyth et al. reported a large series of patients, most of whom had been heavily pretreated with CDK4/6i and chemotherapy, and whom had visceral metastases and very advanced disease and/or were in the later stages of their treatment at the beginning of ABE therapy. Our series comprised only BC patients treated for metastatic disease or a minority of patients in an adjuvant phase, which might explain the higher adherence rate found in our study (92.25% vs. 88.7%).

The identification of potential barriers to adherence represents another critical issue. Stephenson et al. conducted a qualitative study to describe the challenges and obstacles to adherence and persistence to CDK4/6i in 24 patients with advanced BC using a drug claim database over two years [37]. Most patients reported a positive feeling concerning the ease of home administration and the ability to cope with potential side effects. A strong positive relationship with the managing oncologist and trust in the expected benefits were among the significant factors contributing to adherence and persistence. A multifactorial intervention, including accurate patient education, evidence-based strategies for symptom management, and easy communication with providers, may contribute to ensuring adherence. US investigators conducted a qualitative analysis to identify the barriers to and facilitators of adherence to CDK4/6i [38,39]. The barriers to adherence included a lack of knowledge of its efficacy, side effects, and perceptions of the benefits of CDK4/6i. Adequate communication with the healthcare team and caregiver support were also significant determinants of adherence. Financial issues were less frequent but of great concern. Recently, Goetz et al. reported the impact of dose reductions on the efficacy of adjuvant abemaciclib for patients with high-risk early breast cancer [39], showing that the proper management of toxicity and drug reduction as per protocol do not affect efficacy. Japanese investigators reported that a low baseline weight (<54 kg), bone metastases, and hemoglobin level ≤12.4 g/dL were independent predictors of abemaciclib discontinuation for any reason, and that gastrointestinal side effects were the main reason for discontinuation [40].

Our work has some limitations. The measurement of adherence by PDC may not be entirely reliable, as already stated in the medical literature since there is no definitive evidence that the patients had taken oral medication. Second, the reported data came from a group of investigators working in high-volume centers who are highly experienced in managing patients treated with CDK4/6i. Therefore, the adherence and dose intensity data may be optimistic compared to centers with lower experience. Third, the patient sample size may not reflect the reality in all settings, even if the data mirror real-life practice.

## 5. Conclusions

Medical oncologists’ primary concerns regarding oral anti-cancer agent prescription should be related to difficulties related to patient’s understanding and adherence to recommended regimens [41,42]. Precision medicine, which focuses on treating each patient according to the biological features of their disease, has become increasingly prevalent in oncology. Similarly, oncologists can improve precision treatment by identifying the traits, preferences, and needs of each patient in the context of oral anticancer medications.

## Figures and Tables

**Table 1:** Main clinical and treatment characteristics of patients.

Parameters		Number of Patients (Percent)
Number of patients		100 (100%)
Median age (years, range)		62 (37–75)
Median performance status (range)		2 (0*–*2)
	ECOG 0	66 (66%)
	ECOG 1	31 (31%)
	ECOG 2	3 (3%)
Symptoms (yes/no)		17 (17%)/83 (83%)
Dominant disease	visceral	18 (18%)
	bone only	23 (23%)
	soft tissue *	50 (50%)
	other	9 (9%)
Level of education	basal	25 (25%)
	high school	40 (40%)
	university	31 (31%)
	not available	4 (4%)
Socioeconomical status	low	19 (19%)
	average	53 (53%)
	high	23 (23%)
	not available	(5%)
Caregiver	present	94 (94%)
	absent	6 (6%)
Polypharmacy **	0*–*5 drugs	66 (66%)
	>5 drugs	34 (34%)
Anticancer therapy	LHRH	23 (23%)
	abemaciclib	100 (100%)
	AI	63 (63%)
	fulvestrant	27 (27%)
Starting abemaciclib	150 mg b.i.d.	84 (84%)
dose	100 mg b.i.d.	16 (16%)

* Soft tissue: Lymph nodes, skin: ** Polypharmacy: more than five different medications taken for diseases other than cancer.

**Table 2:** Adherence rate, reasons for non-adherence, and dose intensity.

Parameters	Results
N. of patients	100 (100%)
Follow-up	12 months
Adherence rate (mean + SE)	92.25% + 1.939
Non-adherence rate	12.5%
Factor potentially linked to lower adherence (n. of patients, %)	
-Side effects	26 (26%)
-Delayed claims	7 (7%)
-Intercurrent non-cancer-related illnesses	7 (7%)
Programmed dose intensity	
-150 mf b.i.d.	2100 mg/week (100%)
-100 mg b.i.d.	1400 mg/week (100%)
Received dose intensity	
-150 mg b.i.d.	1968 mg/week (82%)
-100 mg b.i.d.	1350 mg/week (96.4%)
Side effects	
-Diarrhea * (grade 2*–*3)	35 (35%)
-Fatigue (grade 2*–*3)	16 (16%)
-Anorexia (grade 2*–*3)	21 (21%)
-Anemia (grade 2*–*3)	23 (23%)
Dose reductions	
-None	77 (77%)
-100 mg bid	21 (21%)
-50 mg bid	3 (03%)

* Grade 2 prolonged.

## Data Availability

The data presented in this study are available upon request from the corresponding author due to privacy and legal aspects.

## Abbreviations

The following abbreviations are used in this manuscript:

ABE	Abemaciclib
CDK4/6i	Cyclin-dependent kinase 4/6 inhibitors
AI	Aromatase inhibitor
RDI	Received dose intensity
